# Advanced Antibacterial Nanocomposite Fibers for Biomedical Applications

**DOI:** 10.3390/pharmaceutics18060711

**Published:** 2026-06-09

**Authors:** Francisca Acevedo, Manuel Azocar, Eulàlia Sans-Serramitjana, Jeyson Hermosilla, Felipe Gálvez-Jirón, Denisse Bravo, Dayaimi Gonzalez, Gabriela Guajardo, Cristóbal Guajardo, Rodrigo Navia

**Affiliations:** 1 Millennium Nucleus Bioproducts, Genomics and Environmental Microbiology (BioGEM), Avenida España 1680, Valparaíso 2390123, Chile; eulalia.sans@ufrontera.cl (E.S.-S.); jeyson.hermosilla@ufrontera.cl (J.H.); felipe.galvez.j@gmail.com (F.G.-J.); dayigore@gmail.com (D.G.); gabriela.guajardo@ufrontera.cl (G.G.); c.guajardo02@ufromail.cl (C.G.); rodrigo.navia@uss.cl (R.N.); 2 Department of Basic Sciences, Faculty of Medicine, Universidad de La Frontera, Avenida Francisco Salazar 1145, Temuco 4780000, Chile; 3 Center of Excellence in Translational Medicine (CEMT), and Scientific and Technological Bioresource Nucleus (BIOREN), Faculty of Medicine, Universidad de La Frontera, Avenida Francisco Salazar 1145, Temuco 4780000, Chile; 4 Center for Soft Matter Research, SMAT-C, Faculty of Chemistry and Biology, Universidad de Santiago de Chile, Avenida Ecuador, Estación Central, Santiago 9170124, Chile; manuel.azocar@usach.cl; 5 Department of Integral Adult Dentistry, Faculty of Dentistry, Universidad de La Frontera, P.O. Box 54-D, Temuco 4811230, Chile; 6 Doctoral Program in Sciences, Mention in Applied Cell and Molecular Biology, Universidad de La Frontera, Avenida Francisco Salazar 1145, Temuco 4780000, Chile; 7 Microbial Interactions Laboratory, Faculty of Dentistry, Universidad Andrés Bello, Santiago 8370146, Chile; denisse.bravo@unab.cl; 8 Doctoral Program in Natural Resources Sciences, Universidad de La Frontera, Avenida Francisco Salazar 1145, Temuco 4780000, Chile; 9 Biochemistry Program, Faculty of Engineering and Sciences, Universidad de La Frontera, Avenida Francisco Salazar 1145, Temuco 4780000, Chile; 10 Faculty of Engineering, Universidad San Sebastián, Bellavista 7, Santiago 7510158, Chile; 11 Center for Advancing agri-Food System Transformation, Santiago 7500000, Chile

**Keywords:** ligand-stabilized silver nanoparticles, electrospun PCL/PEO composites, nanoparticle-functionalized nanomaterials, biofilm-associated infections, antimicrobial biomaterials

## Abstract

**Background/Objectives:** Wound infections represent a major clinical challenge due to their polymicrobial nature, biofilm formation, and increasing antimicrobial resistance, which compromise conventional treatments. This study aimed to develop and evaluate ligand-stabilized silver nanoparticles (AgNPs) with improved antimicrobial activity and cytocompatibility, and to investigate their incorporation into electrospun nanofibers for wound management. **Methods:** Four AgNP formulations stabilized with citrate, cysteine, ketorolac, and diclofenac were synthesized via chemical reduction. Physicochemical characterization included surface plasmon resonance and zeta potential measurements. Antimicrobial activity was assessed through minimum inhibitory concentration (MIC) and bactericidal assays against Gram-positive, Gram-negative, and fungal strains. Toxicity was evaluated using the HET-CAM assay, while cytocompatibility was determined in fibroblasts, MG-63 cells, and mesenchymal stem cells. Diclofenac-stabilized AgNPs were incorporated into electrospun PCL/PEO nanofibers to generate a functional nanocomposite system. **Results:** All AgNPs exhibited a characteristic SPR at ~400 nm and high colloidal stability. Diclofenac-stabilized AgNPs (dc-AgNPs) showed the highest antimicrobial activity, with MIC values of 18.8 mg/L against *Staphylococcus aureus* and *Pseudomonas aeruginosa*, and 4.7 mg/L against *Candida albicans*, along with strong bactericidal effects. HET-CAM assays indicated negligible irritation at concentrations up to 75 mg/L. Cytocompatibility results revealed a dose-dependent response, with fibroblasts being more sensitive. Electrospun nanofibers loaded with dc-AgNPs achieved a 2.6 log reduction against *Streptococcus mutans* and moderate reductions (0.4–0.7 log) against other pathogens. **Conclusions:** Ligand engineering critically influences the antimicrobial efficacy and biocompatibility of AgNPs. The incorporation of dc-AgNPs into electrospun nanofibers represents a promising approach for treating biofilm-associated wound infections.

## 1. Introduction

Wound infections remain a major global health challenge, affecting millions of individuals and significantly impairing the healing process. The uncontrolled proliferation of microorganisms at the injury site interferes with tissue regeneration and often leads to chronic infections requiring prolonged antibiotic therapy (typically 6–12 weeks). However, these treatments frequently fail to achieve complete resolution, with recurrence occurring after antibiotic withdrawal, primarily due to biofilm persistence [1,2,3].

Current evidence indicates that wound infections are not caused by a single pathogen but by highly structured polymicrobial communities, in which anaerobic bacteria play a critical and often underestimated role. These microorganisms colonize hypoxic niches within wounds, contributing to biofilm maturation, interspecies cooperation, and antimicrobial tolerance, thereby promoting persistent infections and poor clinical outcomes [4,5,6]. Within these biofilms, interactions between aerobic and anaerobic species exacerbate pathogenicity. Facultative anaerobes such as *Staphylococcus aureus* generate hypoxic microenvironments that favor strict anaerobes, while metabolic cross-feeding enhances community stability and resistance [6]. In addition, pathogens such as *Pseudomonas aeruginosa* exhibit intrinsic resistance to multiple antibiotic classes [7,8]. Likewise, *Staphylococcus* species, particularly *S. aureus* and *S. epidermidis*, account for approximately 25% of wound isolates and are frequently associated with persistent infections; notably, infections involving methicillin-resistant *S. aureus* (MRSA) may still show partial clinical responses even under suboptimal antibiotic regimens due to the polymicrobial nature of these communities [9]. Furthermore, microorganisms such as *Candida albicans*, *Porphyromonas gingivalis*, and *Fusobacterium nucleatum* further increase the complexity of the wound microbiota. *C. albicans* forms robust, highly resistant biofilms and establishes synergistic interactions with bacteria such as *S. aureus*, promoting mixed infections. Meanwhile, *P. gingivalis* and *F. nucleatum* modulate host immune responses and contribute to tissue degradation through virulence factors [10,11]. Collectively, these microorganisms enhance the resilience and pathogenicity of chronic wound biofilms.

This complex microbial ecology, characterized by polymicrobial interactions, biofilm formation, and antimicrobial resistance, highlights the urgent need for alternative therapeutic strategies capable of effectively targeting infections at the wound site. In this context, advanced wound dressings with intrinsic antimicrobial properties have emerged as promising tools to prevent and control infection while promoting tissue repair. Electrospinning is particularly attractive, as it enables the fabrication of nanofibrous scaffolds that mimic the native extracellular matrix (ECM) and support tissue regeneration [12,13,14], while allowing the incorporation of bioactive agents.

Among these, silver nanoparticles (AgNPs) have gained attention due to their broad-spectrum antibacterial activity, mediated by multimodal mechanisms including membrane disruption, intracellular penetration, reactive oxygen species (ROS) generation, and modulation of cellular pathways [15]. However, their clinical application is limited by potential cytotoxic effects associated with oxidative stress. To overcome this, surface functionalization using capping agents has been employed to enhance nanoparticle stability and reduce toxicity without compromising antimicrobial activity [16,17,18]. These stabilizers, such as dextrans, polyvinylpyrrolidone (PVP), trisodium citrate, peptides, and graphene oxide, play a key role in controlling nanoparticle size, preventing aggregation, and modulating biological interactions [19,20,21].

Therefore, this study aimed to evaluate the antibacterial activity of ligand-stabilized silver nanoparticles and explore their potential integration into advanced biomaterial systems for biomedical applications.

## 2. Materials and Methods

### 2.1. Materials

All of the chemicals and reagents used in this research were of analytical grade and purchased from Sigma-Aldrich (St. Louis, MO, USA) or Merck KGaA (Darmstadt, Germany). Silver nitrate (AgNO_3_, 99.8%, Sigma-Aldrich), NaBH_4_ (97%), sodium citrate, l-cysteine, ketorolac and diclofenac were used. Milli-Q water was used to prepare all the samples.

### 2.2. Synthesis and Physicochemical Characterization of Ligand-Stabilized Silver Nanoparticles

Four variants of silver nanoparticles (AgNPs) were synthesized by chemical reduction using sodium borohydride (NaBH_4_) as the reducing agent in a 300 mg/L AgNO_3_ solution. The synthesis was carried out at room temperature in the presence of sodium citrate (NaCitr), cysteine (Cyst), ketorolac (kt) or diclofenac (dc) as stabilizing agents.

The formation and temporal stability of the nanoparticles were monitored by UV–visible spectroscopy using a Shimadzu UV-1800 spectrophotometer (Shimadzu Corporation, Kyoto, Japan). Spectra were recorded in the 350–700 nm range using 3 mL quartz cuvettes containing 0.5 mL of AgNP suspension diluted with 2 mL of Milli-Q water. In all samples, a characteristic surface plasmon resonance (SPR) band was observed at approximately 400 nm, confirming the formation of AgNPs.

The size and morphology of the nanoparticles were characterized by transmission electron microscopy (TEM) using a HITACHI HT7700 microscope (Hitachi High-Tech Corporation, Tokyo, Japan). Samples were prepared by depositing the nanoparticle suspension onto 400-mesh copper grids and allowing them to dry at room temperature (25 °C).

The surface charge (zeta potential, ζ) of the nanoparticles was determined by dynamic light scattering using a Zetasizer Nano ZS instrument equipped with a DTS1070 cell (Malvern Instruments, Malvern, UK).

### 2.3. Antimicrobial Susceptibility of Planktonic Bacteria

In vitro microbiological assays were performed to determine the minimum inhibitory concentration (MIC) and minimum bactericidal concentration (MBC) of the ligand-stabilized silver nanoparticles against aerobic and anaerobic bacterial strains (*E. coli* ATCC 25922, *S. aureus* ATCC 25923, *P. aeruginosa* ATCC 27853, *S. mutans* ATCC 25174, *Porphyromonas gingivalis* ATCC 53978, *Fusobacterium nucleatum* ATCC 10953) and one yeast strain (*C. albicans)* obtained from the Bioprocesses and Bioseparations Laboratory, Universidad de La Frontera, Temuco, Chile, by using the broth microdilution method in accordance with the Clinical Laboratory Standards Institute (CLSI) M100, 35th edition (2025) [22]. For antimicrobial susceptibility testing, culture media were selected according to the microorganism type. Mueller–Hinton broth (MHB) was used for aerobic and facultative bacteria, Sabouraud dextrose broth was used for *C. albicans* and brain heart infusion (BHI) broth was used for anaerobic bacteria in MIC assays. For MBC determination, Mueller–Hinton agar was used for aerobic/facultative bacteria, Sabouraud dextrose agar for *C. albicans* and BHI agar for anaerobic bacteria. Serial dilutions of AgNPs were prepared in the corresponding culture media in 96-well microplates, starting from an initial nanoparticle concentration of 300 mg/L, filling consecutive wells to a final volume of 100 μL. Subsequently, 5 μL microorganism suspensions prepared at 1.0 × 10^7^ CFU/mL (bacteria) and 1.0 × 10^5^ CFU/mL (fungus) were added to each well. Microorganism suspensions without nanocomposites were used as controls. Cell densities were estimated by measuring absorbance at 600 nm for bacteria and 620 nm for fungi using an Infinite^®^ M200 PRO microplate reader (Tecan, Männedorf, Switzerland).

The plates were incubated for 16–18 h at 37 °C for bacteria and 43–45 h at 30 °C for fungi. After incubation, MIC values were determined by visual inspection, based on the presence or absence of microbial growth. Wells showing inhibition were selected for MBC determination. For this, 5 μL from the selected wells was plated onto solid media and incubated for 24 h at 37 °C. MBC was defined as the lowest concentration at which no microbial growth was observed. Appropriate medium controls and positive controls were included in all experiments.

### 2.4. Biocompatibility Assays of Ligand-Stabilized Silver Nanoparticles

#### 2.4.1. Cell Culture

MG-63 was osteoblast-like and cultured in α-MEM, and dermal fibroblast (3T3L1/CL-173) was cultured in DMEM medium. Both media were supplemented with 10% (*v*/*v*) fetal bovine serum (HyClone, USA) and 1% (*v*/*v*) penicillin and streptomycin (HyClone, USA). The cells were maintained at a temperature of 37 °C in a humidified atmosphere with 95% humidity and 5% CO_2_. When the flasks reached approximately 90% confluency, indicating that the cells had grown and covered most of the culture surface, they were subcultured. To detach the cells from the tissue culture surface, they were exposed to 0.25% (*w*/*v*) trypsin-EDTA solution (Corning, Singapore) for three minutes.

#### 2.4.2. Proliferation Analysis

Cell proliferation in the presence of nanocomposites (0.146–150 µg/mL) was evaluated using MTT assay, following the manufacturer’s recommendations. Subsequently, nanocomposites were placed into 24-well microplates. Each well was seeded with 10,000 cells in 500 µL of culture media of MG-63 or fibroblast cell lines and each cell line was incubated at 37 °C. The experiments were conducted in triplicate for each treatment and control group. Cell viability was assessed after 24 h of incubation. Following incubation, the medium was removed and replaced with 150 µL of MTT reagent per well.

### 2.5. Hen’s Egg Chorioallantoic Membrane (HET-CAM)

The HET-CAM methodology was used to evaluate the toxicity of ligand-stabilized silver nanoparticles on the chorioallantoic membrane of chicken eggs. The eggs were incubated for 9 days in an incubation chamber at 37 °C and 60% humidity. To access the chorioallantoic membrane, a small hole was made in the eggshell at the wide part (where the air chamber is located), taking great care not to damage the inner membrane. Then, the inner membrane was completely moistened with 0.9% (*w*/*v*) NaCl. The egg was placed back in the incubator for a maximum of 30 min. Then, the membrane was carefully removed using tweezers. Then, 300 µL of dc-AgNPs at different concentrations (4.65, 18.75, 37.5 and 75.0 mg/L), positive control, or negative control was added, as appropriate; 0.1 N NaOH (sodium hydroxide) was used as a positive control, and 0.9% (*w*/*v*) NaCl (sodium chloride) as a negative control. The test was performed in duplicate.

Finally, the membrane was observed for 5 min (300 s) to identify the possible effects of hemorrhage (bleeding from the vessels), vascular lysis (disintegration of blood vessels) and coagulation (denaturation of intra- and extra-vascular proteins). Based on the measured times, the irritation index (IS, irritation score) was calculated using the following equation (Equation (1)):

(1) $$IS=\left(\frac{\;301-hemorrhage\;time}{300}\right)*5\;+\;\left(\frac{\;301-lisis\;time}{300}\right)*7\;+\;\left(\frac{\;301-coagulation\;time}{300}\right)*9$$

The test will be considered acceptable if the negative and positive controls induce a non-irritating and severely irritating response, respectively (Table 1).

### 2.6. Fabrication of PCL/PEO-AgNP Electrospun Nanofibers

Electrospun fibers were produced using a NEU-BM electrospinning system (Tong Li Tech, Shenzhen, China) under ambient conditions (20 °C and 50% relative humidity). The electrospinning parameters were optimized to obtain nanocomposite nanofibrous mats using an applied voltage of 25 kV, a solution flow rate of 2 mL h^−1^, a tip-to-collector distance of 20 cm, and a collector rotation speed of 50 rpm, following a previously reported protocol [25].

Two polymer solutions were prepared: 14% (*w*/*v*) PCL in chloroform and 10% (*w*/*v*) PEO in aqueous solution of dc-AgNPs (75 mg/L). The membrane was fabricated by simultaneous electrospinning of both polymeric solutions for 12 min, followed by an additional 3 min of electrospinning with the PCL solution alone. This final PCL deposition step was included to reinforce the hydrophobic character and structural stability of the membrane surface. As a negative control, membranes were produced under the same electrospinning conditions, but using PEO solution without dc-AgNPs.

### 2.7. Determination of Physicochemical Properties of PCL/PEO-AgNP Electrospun Nanofibers

#### 2.7.1. Morphology and Fiber Diameter Analysis

Fiber morphology and diameter were examined by scanning electron microscopy (SEM; Hitachi SU2500, Hitachi High-Tech, Japan). Prior to imaging, samples (*n* = 3) were sputter-coated with an approximately 8 nm gold layer using an AUTO 108 argon sputter coater (Cressington Scientific Instruments Ltd., Watford, UK). SEM micrographs were analyzed using ImageJ software (National Institutes of Health, Bethesda, MD, USA; https://imagej.nih.gov/ij/, accessed on 2 June 2026) to determine fiber diameter distribution and morphological characteristics. The morphology index (MI) was used as a semi-quantitative parameter to assess fiber homogeneity and morphological quality from SEM micrographs, where values closer to 1 indicate more uniform and defect-free fibers. The MI calculation was performed according to the methodology previously described by Hermosilla et al. [25]. 

#### 2.7.2. Proliferation Analysis

Cell proliferation in the presence of nanocomposites (0.146–150 µg/mL) was evaluated using MTT assay, following the manufacturer’s recommendations. Subsequently, nanocomposites were placed into 24-well microplates. Each well was seeded with 10,000 cells in 500 µL of culture media of MG-63 or fibroblast cell lines and each cell line was incubated at 37 °C. The experiments were conducted in triplicate for each treatment and control group. Cell viability was assessed after 24 h of incubation. Following incubation, the medium was removed and replaced with 150 µL of MTT reagent per well. For cellular assays, membrane specimens of approximately 1 × 1 cm were used, corresponding to an estimated dry weight of approximately 1 mg per sample.

#### 2.7.3. Thermal Properties

Thermal properties were analyzed by TGA and DSC using an STA 6000 system (PerkinElmer Inc., Waltham, MA, USA). For TGA, approximately 20 mg of sample was heated from 25 to 600 °C at 15 °C min^−1^ under nitrogen flow. The degradation temperature (T_d_) was determined from the TGA/DTG profile as the temperature of the main degradation event, corresponding to the maximum mass-loss rate. For DSC, the melting temperature (T_m_) and melting enthalpy (ΔH_m_) were obtained from the thermograms. The crystallinity degree (C_x_) was calculated as (Equation (2))

Cₓ (%) = (ΔHₘ/ΔHₘ^0^) × 100 (2)

with ΔH_m_^0^ values of 139.3 J g^−1^ for PCL and 203 J g^−1^ for PEO. For PCL/PEO–dc AgNP fibers, C_x_ was reported as apparent crystallinity using the PCL reference enthalpy.

#### 2.7.4. FTIR Spectra Analyses

The chemical structure of the nanofibers was analyzed using Fourier-transform infrared (FTIR) spectrometry. The FTIR measurements were performed using a Cary 630 FTIR Spectrometer from Agilent Technologies Inc. (Danbury, CT, USA). The software Resolution Pro version 2.20.06 was employed for data analysis. A scanning range of 4000–600 cm^−1^ was used to obtain the FTIR spectra.

### 2.8. Mechanical Characterization

Tensile properties of the electrospun nanofibrous mats were evaluated using rectangular specimens (4.0 × 1.0 cm). Mechanical testing was performed using a CT3-10KG digital texture analyzer (Brookfield Engineering Laboratories, Middleboro, MA, USA). Samples were mounted between mechanical grips with an initial gauge length of 8 mm and tested at a crosshead speed of 0.1 mm s^−1^ until failure. From the resulting stress–strain curves, tensile modulus, tensile strength, and elongation at break were calculated. All measurements were conducted at room temperature, and each sample was tested in quadruplicate.

### 2.9. Antimicrobial Analysis of PCL/PEO-dc-AgNP Electrospun Nanofibers

#### 2.9.1. ASTM E2149 Test: “Dynamic Shake Flask Test”

The ASTM E2149 test quantitatively evaluates the antimicrobial effectiveness of samples under dynamic contact conditions. The tests were performed using the methodology by Song et al. [26] with slight modifications adapted to electrospun samples. Both Gram-positive *S. aureus* (ATCC 25923) and Gram-negative *P. aeruginosa* (ATCC 27853) bacteria were used. The pre-inoculums of bacteria were prepared in tryptic soy broth (TSB) (Merck KGaA, Darmstadt, Germany) and were incubated by 10–12 h at 37 °C and 120 rpm. Next to each, bacterial inoculum was concentrated by centrifugation (4000× *g* rpm and 10× *g* min), the supernatant was eliminated and the bacteria was washed with sterile phosphate-buffered saline (PBS). Then, the concentration of each bacterium was adjusted to 1.0 × 10^7^ CFU/mL. For the shake flask assay, 2 × 1 cm samples were used, corresponding to an approximate dry weight of 2 mg per specimen. The samples were inoculated in 5 mL of bacterial suspension for 5 h at 37 °C and 120 rpm. Subsequently, 200 µL aliquots were obtained in triplicate from each sample and added in 96-well plates. Serial dilutions (1:10) were then made from each of the samples using PBS for dilution. Finally, both the samples and the dilutions were cultured on agar plates for 24 h at 37 °C to determine the number of colony-forming units (CFU/mL). Results were expressed as log reduction, calculated as the ratio of the number of surviving bacterial colonies present on tryptic soy agar (TSA) plates (Merck) after contact with samples (Equation (3)):

*log_reduction_ = log_Ø_ − log_s_* (3)

where *log_Ø_* is the average of CFU/mL control log, and *log_s_* is the average of sample CFU/mL log. Studies were performed in triplicate. For the *C. albicans* (ATCC 90028) the methodology is the same, except for the incubated temperature, where 37 °C is switched for 30 °C.

#### 2.9.2. Test Method 100-TM100: “Contact Killing Test”

American Association of Textile Chemists and Colorists (AATCC) Test Method 100-TM100 (AATCC, 2012) was adapted by Padrão et al. [27] to assess the contact antimicrobial efficacy of samples, called the contact killing test. This methodology was adapted for nanocomposite nanofibers, being evaluated against *S. aureus*, *P. aeruginosa*, and *C. albicans*. For the contact killing assay, 1 × 1 cm samples were used, corresponding to approximately 1 mg per specimen. Briefly, electrospun fibers of 1 × 1 cm were placed in Petri plates and inoculated with 20 µL of microorganisms at a concentration of 1.0 × 10^7^ CFU/mL per 1 h of incubation at room temperature. Then, the samples were moved to 15 mL tubes and covered with 5 mL of PBS, and stirred for 1 min, to homogenize. Next, the CFU/mL was determined in Mueller–Hinton (MH) agar Petri plates incubated for 24 h at 37 °C. The log reduction was determined for Equation 1. Studies were performed in triplicate. For the *C. albicans* the methodology is the same, except for the incubated temperature, where 37 °C is switched for 30 °C.

## 3. Results and Discussion

### 3.1. Synthesis and Physicochemical Characterization of Ligand-Stabilized Silver Nanoparticles

Four ligand-stabilized silver nanoparticles were successfully synthesized by chemical reduction: sodium citrate–AgNPs (NaCitr-AgNPs), cysteine–AgNPs (cyst-AgNPs), ketorolac–AgNPs (kt-AgNPs) and diclofenac–AgNPs (dc-AgNPs).

**Figure 1:**
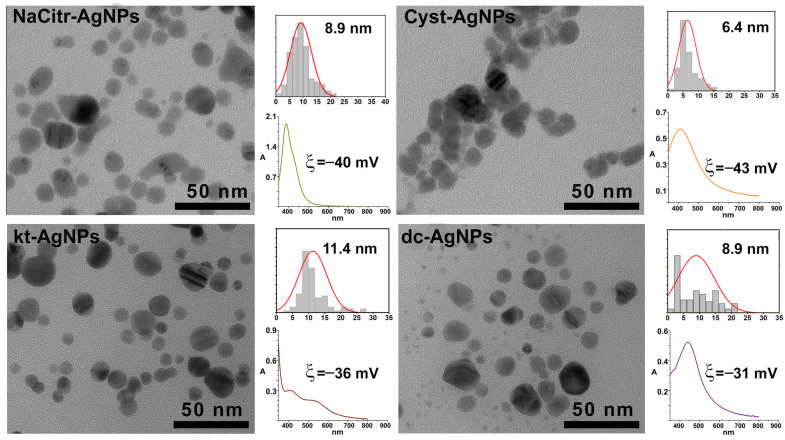
Physicochemical characterization of ligand-stabilized silver nanoparticles (NaCitr-AgNPs, Cyst-AgNPs, kt-AgNPs, dc-AgNPs) by UV–Vis spectra, TEM micrographs and Zeta potential (ζ) values.

The formation and evolution of the nanoparticles were monitored by UV–visible spectroscopy (Figure 1). In all samples, a characteristic surface plasmon resonance (SPR) band was observed at approximately 400 nm, confirming the formation of AgNPs. UV–visible spectra were recorded over several days, revealing high spectral stability of the nanoparticles over time. The colloidal suspensions were subsequently stored in the dark at 4 °C. In the kt-AgNP sample, two distinct absorption bands were detected, the typical SPR band and an additional band around 500 nm, which may be attributed to an interfacial charge transfer (ICT) process. This phenomenon has been associated with nanoparticle oxidation and may indicate a potential increase in toxicity.

Transmission electron microscopy (TEM) was employed to analyze the morphology and size distribution of the ligand-stabilized silver nanoparticles (Figure 1). Cyst-AgNPs displayed predominantly spherical morphology with a relatively narrow size distribution (1–15 nm), although some aggregation was observed. This behavior suggests strong interaction between the thiol group of cysteine and the nanoparticle surface, leaving the carboxyl and amino groups exposed for further interactions [28]. In contrast, NaCitr-AgNPs exhibited slightly larger particle sizes, occasional anisotropic morphologies, and reduced aggregation, indicating weaker surface interactions compared to cysteine. The dc-AgNPs showed a broader distribution of quasi-spherical and irregularly shaped nanoparticles, possibly reflecting lower size control or post-synthesis Ostwald ripening. A similar trend was observed for kt-AgNPs; however, larger particles were generally detected (average maximum size ≈ 11.4 nm), with few nanoparticles below 5 nm. Overall, the nature of the stabilizing ligand, including anti-inflammatory drugs (ketorolac and diclofenac) as well as cysteine and citrate, significantly influenced nanoparticle morphology, size distribution, and dispersion state, highlighting the role of ligand–metal interactions during nucleation and growth of Ag^0^ nanoparticles.

Zeta potential (ζ) measurements of all ligand-stabilized silver nanoparticles showed negative values ranging from −31 to −43 mV, indicating good colloidal stability in aqueous media (Figure 1).

### 3.2. Antimicrobial Susceptibility of Planktonic Bacteria of Ligand-Stabilized Silver Nanoparticles

As presented in Table 2, dc-AgNPs exhibited the lowest MIC values across the most tested microorganisms. Their most pronounced activity was observed against *S. aureus* and *P. aeruginosa* (MIC: 18.8 mg/L), with exceptionally high efficacy against *C. albicans* (MIC: 4.7 mg/L). Furthermore, effective inhibition was noted for *S. mutans* (37.5 mg/L) and *E. coli* (75 mg/L). Collectively, these data position the dc-AgNPs as the most potent agents within this microbial panel.

These findings are consistent with existing literature that highlights the bactericidal properties of diclofenac against pathogens such as *S. aureus*, *E. coli*, *Listeria monocytogenes*, and *Mycobacterium* spp. Its mechanisms of action include the inhibition of DNA synthesis in *E. coli* and *L. monocytogenes*, providing protection against infections like listeriosis and tuberculosis in murine models [29,30].

Cyst-AgNPs demonstrated moderate to high activity (MICs ranging from 75 to 150 mg/L) against most bacteria and an MIC of 37.5 mg/L against *C. albicans*. However, their potency was surpassed by diclofenac and other formulations. Cysteine and its derivatives possess inherent antimicrobial properties, both independently and synergistically with antibiotics. The antimicrobial efficacy of cysteine is also noted to be stereo-specific (L-cysteine vs. D-cysteine), with proposed mechanisms involving the disruption of microbial cell membrane integrity [31,32].

Kt-AgNPs showed effectiveness against *P. aeruginosa* (37.5 mg/L) and *C. albicans* (9.4 mg/L). Conversely, higher concentrations (150 mg/L) were required for activity against *S. aureus* and *S. mutans.* While ketorolac, a nonsteroidal anti-inflammatory drug (NSAID), has been reported to exhibit mild to moderate antimicrobial activity against *S. aureus* and *E. coli*, it generally lacks efficacy against fungi such as *C. albicans* [33] and often performs optimally in combination with other agents like gentamicin [34]. The observed inhibition of *C. albicans* and *P. aeruginosa* by kt-AgNPs in this study suggests a synergistic effect between AgNPs and ketorolac that warrants further investigation, as it is not extensively documented in the current literature.

AgNPs synthesized with sodium citrate as a reducing and stabilizing agent exhibited comparatively higher MICs, notably for *P. aeruginosa* and *E. coli* (300 mg/L), indicating reduced effectiveness against these strains. Moderate activity was maintained against *S. aureus* (75 mg/L) and *C. albicans* (75 mg/L). Sodium citrate is recognized as a food additive capable of inhibiting biofilms and reducing bacterial virulence in strains such as *Serratia marcescens* and *P. aeruginosa*, and as a bacteriostatic agent against *E. coli* [35].

In summary, the MIC results strongly indicate that the choice of stabilizer profoundly influences the antimicrobial activity of silver nanoparticles. Among the formulations tested, dc-AgNPs demonstrated the most favorable combination of low MIC values and a broad spectrum of antimicrobial action.

Table 3 shows the MBC results for the evaluated nanocomposites. Dc-AgNPs demonstrated the highest bactericidal activity across all tested microorganisms, requiring only 75 mg/L to eliminate *S. aureus*, *P. aeruginosa*, and *E. coli*, and 150 mg/L for *S. mutans*. Kt-AgNPs ranked second in bactericidal efficacy, with an MBC of 150 mg/L for *S. aureus*, *P. aeruginosa*, and *E. coli*, though 300 mg/L was needed for *S. mutans*. In contrast, Cyst-AgNPs and NaCitr-AgNPs failed to eliminate *S. aureus*, *P. aeruginosa*, or *E. coli* at concentrations up to 300 mg/L (reported as >300 mg/L). However, both achieved an MBC of 300 mg/L against *S. mutans*. Regarding fungicidal activity against *C. albicans*, Dc-AgNPs showed the most potent effect (18.75 mg/L), significantly lower than the other nanocomposites. kc-AgNPs exhibited intermediate activity (75 mg/L), while Cyst-AgNPs and NaCitr-AgNPs displayed lower activities (300 mg/L).

In conclusion, the MBC results for both bacteria and fungi confirm that dc-AgNPs are the most promising option within this set for broad-spectrum antimicrobial action.

### 3.3. Biocompatibility Assays of Ligand-Stabilized Silver Nanoparticles

**Figure 2:**
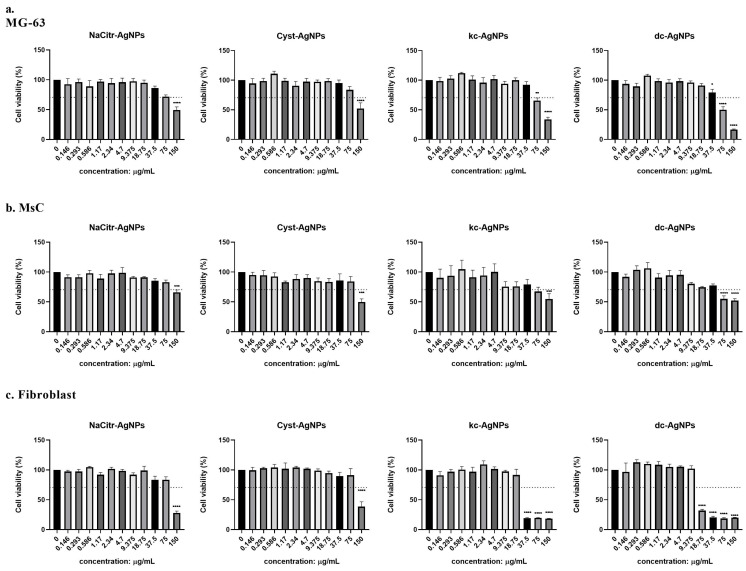
Effects of AgNPs on the viability of MG-63, MsC and fibroblast cells. The viability of (**a**) MG-63 (**b**) MsC and (**c**) Fibroblast cells treated with different concentrations of NaCitr-AgNpsCyst-AgNPs, kc-AgNPs and dc-AgNPs for 24 h. Data are expressed as mean ± SEM. The dotted line represents the 70% cell viability threshold according to ISO 10993-5. Statistical differences are shown compared with non-treated cells. Statistical differences were processed with a one-way ANOVA with Tukey’s post hoc test (* *p* < 0.05, ** *p* < 0.01, *** *p* < 0.001, and **** *p* < 0.0001).

The biocompatibility of the four ligand-stabilized silver nanoparticles was evaluated using the MTT assay after 24 h of exposure in MG-63 cells, mesenchymal stem cells (MSCs), and fibroblasts, as shown in Figure 2. For a better understanding, the results are discussed according to the lowest concentration at which each formulation induced a significant reduction in cell proliferation.

Overall, the cytotoxic response depended on both the stabilizing ligand and the cell type. MG-63 cells showed relatively high tolerance to NaCitr-AgNPs and Cys-AgNPs, with significant reductions in proliferation observed only at the highest tested concentration, 150 µg/mL. In contrast, the drug-stabilized formulations induced cytotoxic effects at lower concentrations. Specifically, kc-AgNPs significantly reduced MG-63 cell proliferation from 75 µg/mL, whereas dc-AgNPs induced a significant decrease from 37.5 µg/mL, indicating a stronger concentration-dependent effect for the diclofenac-stabilized formulation.

A similar, although less pronounced, pattern was observed in MSCs. NaCitr-AgNPs, Cys-AgNPs, and kc-AgNPs significantly affected MSC proliferation only at 150 µg/mL, suggesting greater tolerance of this cell type to most formulations. However, dc-AgNPs reduced MSC proliferation from 75 µg/mL, supporting the comparatively higher cytotoxic potential of the diclofenac-stabilized nanoparticles.

Fibroblasts were the most sensitive cell type evaluated. Whereas NaCitr-AgNPs significantly reduced fibroblast proliferation only at 150 µg/mL, Cys-AgNPs and kc-AgNPs induced significant effects from 37.5 µg/mL. Notably, dc-AgNPs significantly reduced fibroblast proliferation from 18.75 µg/mL, indicating that this formulation exerted the strongest cytotoxic effect among the tested nanoparticles. These results suggest an overall sensitivity trend of fibroblasts > MG-63 cells ≈ MSCs, particularly in response to the drug-stabilized AgNPs.

Taken together, these findings indicate that NaCitr-AgNPs and Cys-AgNPs exhibited the most favorable biocompatibility profiles, as their effects were mainly restricted to the highest tested concentration. Conversely, kc-AgNPs and, more markedly, dc-AgNPs showed greater cytotoxicity at lower concentrations, especially in fibroblasts. Although IC_50_ values were not determined in this study, the concentration-threshold analysis provides a clear comparative overview of the biological response elicited by each nanoparticle formulation.

The precise mechanisms underlying AgNP-induced cytotoxicity remain incompletely understood. However, several processes have been proposed, including Ag^+^ ion release, interactions with membrane proteins and nucleic acids, lysosomal membrane disruption, oxidative stress, and increased membrane permeability [36]. Our findings are consistent with previous studies indicating that MSCs generally exhibit greater tolerance to AgNP-based materials, whereas fibroblasts tend to be more sensitive [37,38]. In this context, the stabilizing ligand appears to play an important role in modulating the biological response, potentially by influencing nanoparticle stability, Ag^+^ release, and cell–nanomaterial interactions.

### 3.4. Hen’s Egg Chorioallantoic Membrane (HET-CAM)

**Figure 3:**
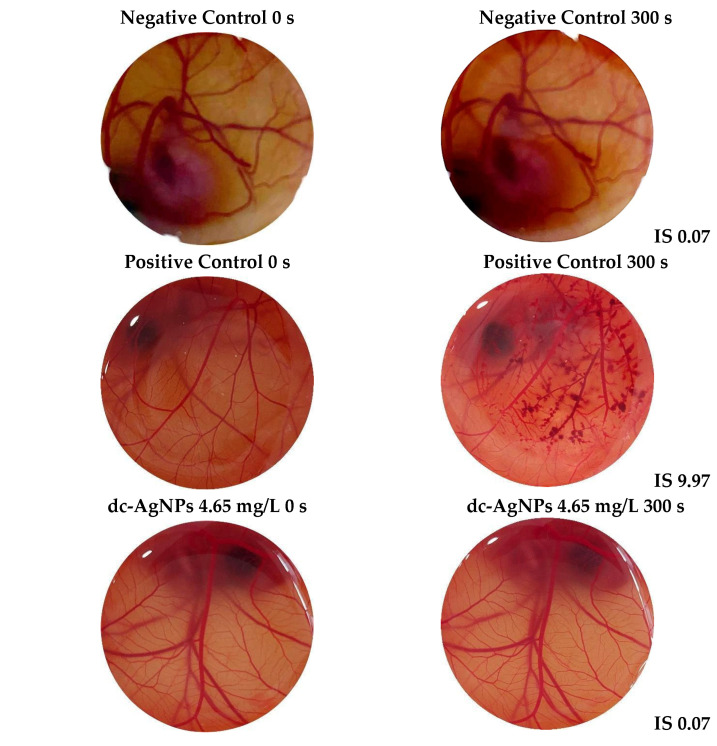

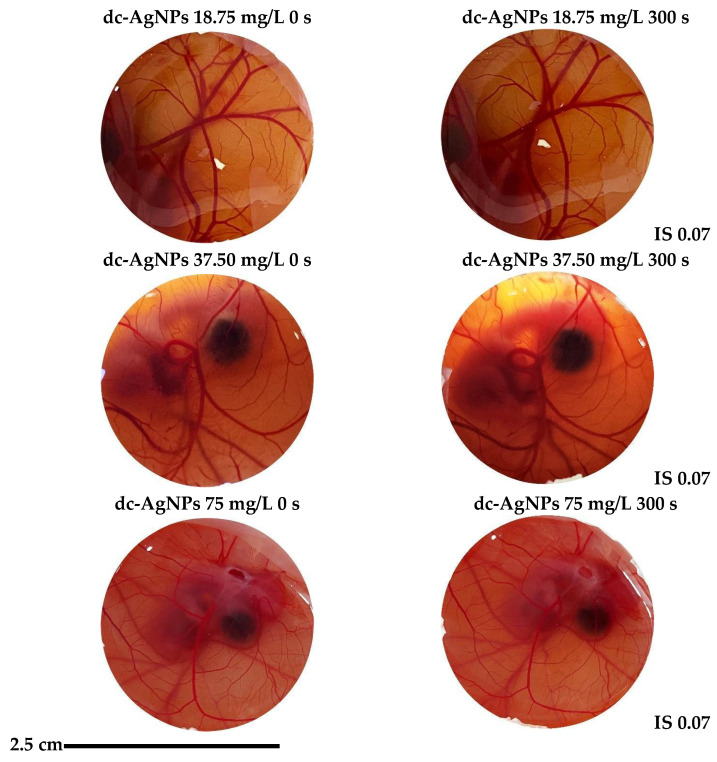
Photographs of HET-CAM test results of dc-AgNPs (4.65; 18.75; 37.5 and 75 mg/L). The values in red indicate the irritation index. Each circular panel has a diameter of 2.5 cm.

The HET-CAM assay was used to evaluate the irritating potential of dc-AgNPs at different concentrations (4.65, 18.75, 37.5 and 75.0 mg/L). As a reference, a negative control (sodium chloride) and a positive control (sodium hydroxide) were included. The results obtained are shown in Figure 3. The dc-AgNP formulations at all their evaluated concentrations presented an IS of 0.07, a value identical to that presented in the negative control, which classifies them within the “Nonirritant” category according to the ranges of the HET-CAM assay. On the other hand, the positive control showed an IS of 9.97, indicating strong or severe irritation, validating the sensitivity of the assay. These results suggest that the evaluated nanoparticles do not generate significant irritation in the chorioallantoic membrane of the egg. These results are consistent with published studies, which establish that AgNPs coated with different substances did not promote vasoconstriction, hemorrhage, or coagulation in the chorioallantoic membrane, indicating that they are non-irritating and have low toxicity. Xie et al. [39] synthesized AgNPs coated with trisodium citrate (TSC-AgNP) or polyvinylpyrrolidone (PVP-AgNP) and evaluated the irritating potential by HET-CAM. The results obtained when evaluating AgNPs at 100 mg/L concentration were non-irritant. Freire et al. [40] evaluated AgNPs stabilized with chitosan at different concentrations, and the nanoparticles did not show irritating potential. Li et al. [41] tested AgNPs with chitosan and alginic acid (CS/AL-AgNPs) as well as chitosan and alginic acid with tetracycline hydrochloride (CS/AL-TCH); the results showed that the formulations with AgNPs were not irritating.

### 3.5. Fabrication of PCL/PEO-dcAgNP Electrospun Nanofibers

The electrospun nanofibers exhibited a hierarchically organized fibrous architecture arising from the intrinsic physicochemical differences between the PCL and PEO–dc-AgNP systems processed under identical electrospinning conditions (25 kV, 2 mL/h, 20 cm). The resulting membranes had approximate dimensions of 15 × 6 cm, a thickness of 25 µm, and a total dry weight of approximately 90 mg, corresponding to an estimated mass per area of 1 mg cm^−2^.

#### 3.5.1. Determination of Physicochemical Properties of PCL/PEO-dc-AgNP Electrospun Nanofibers

Quantitative SEM analysis demonstrated that PCL 14% fibers presented a mean diameter of 3.212 ± 1.577 µm, whereas PEO 10% fibers incorporating diclofenac-coated silver nanoparticles reached significantly smaller diameters of 0.343 ± 0.073 µm (Table 4). Statistical analysis confirmed that this reduction is highly significant (*p* < 0.0001), indicating a pronounced structural distinction between both fiber populations.

The micrometric dimensions and broader diameter distribution observed in PCL fibers suggest moderate jet instability during the elongation and whipping phases, leading to greater heterogeneity along individual filaments. This behavior is reflected in the morphology index (MI = 0.65), which indicates acceptable but comparatively lower uniformity. In contrast, the PEO–dc-AgNP system exhibited a substantially narrower diameter distribution and a higher morphology index (MI = 0.85), consistent with improved fiber homogeneity and reduced structural irregularities. The incorporation of dc-AgNPs likely increased the electrical conductivity of the spinning solution, enhancing surface charge density along the polymer jet. Elevated Coulombic repulsion promotes greater electrohydrodynamic stretching prior to solvent evaporation, resulting in finer and more uniform nanofibers. Importantly, no severe morphological defects were observed, suggesting adequate nanoparticle dispersion within the matrix.

The fiber diameters obtained in this study are consistent with values commonly reported for electrospun fibers based on PCL and PEO. Electrospun PCL fibers typically present diameters ranging from the submicron scale to several micrometers depending on polymer concentration and solvent system, frequently falling within the 1–5 µm range for solutions with high polymer content [25,42,43]. Similarly, electrospun PEO fibers are often reported in the nanometric to submicrometric range (approximately 100–600 nm), particularly when electrospun from aqueous solutions or when conductive additives are incorporated [44,45]. Therefore, the micrometric PCL fibers and the finer PEO-based nanofibers observed here fall within the expected morphological ranges described in the literature for electrospun systems based on these polymers.

**Figure 4:**
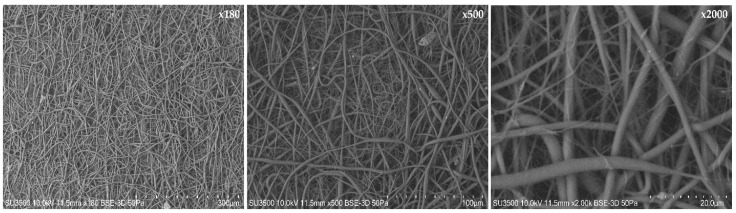
SEM of electrospun PCL/PEO dc–AgNP nanofibers at (×180), (×500) and (×2000) magnifications.

The SEM micrographs of the electrospun PCL/PEO Diclofenac–AgNP nanofibers at different magnifications (Figure 4) reveal a heterogeneous fibrous network composed of two distinct fiber populations. At lower magnification (×180), the membrane exhibits a dense and randomly oriented architecture characteristic of electrospun scaffolds, forming a highly porous and interconnected structure. At intermediate magnification (×500), two clearly distinguishable fiber diameters can be observed within the network. The thicker fibers correspond to the PCL component, which provides the main structural support of the membrane, while the thinner fibers are associated with the PEO matrix containing diclofenac-coated AgNPs. This bimodal fiber distribution is consistent with the multilayer electrospinning strategy used during membrane fabrication. At higher magnification (×2000), the fibers appear smooth and continuous, with no significant bead formation or structural defects. The thinner PEO-based fibers are interwoven between the larger PCL fibers, generating a hierarchical architecture that increases the overall surface area of the membrane. Additionally, small bright domains occasionally observed along the fibers may correspond to nanocomposite regions associated with the incorporated AgNPs. Overall, the SEM analysis confirms the successful formation of an interlaced fibrous network composed of structurally supportive PCL fibers and finer PEO/dc–AgNP fibers, resulting in a porous scaffold morphology suitable for biomedical applications where high surface area and interconnected porosity are desirable.

#### 3.5.2. Elemental Mapping Analysis (SEM–EDS)

**Figure 5:**
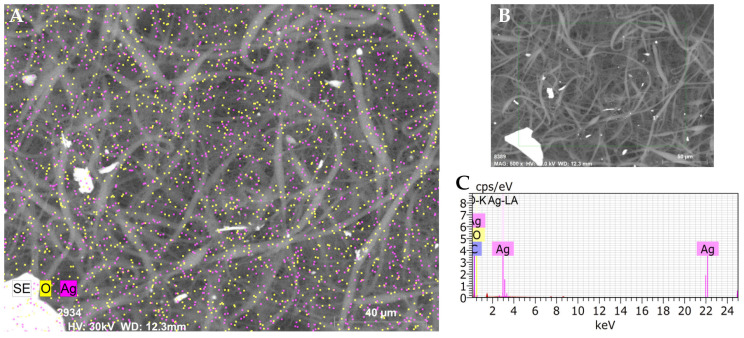
SEM–EDS characterization of electrospun PCL/PEO dc–AgNP nanofibers: (**A**) elemental mapping showing the homogeneous distribution of silver (Ag-Lα) and oxygen (O-K) within the fibrous network; (**B**) SEM micrograph of the electrospun membrane; and (**C**) EDS spectrum confirming the presence of Ag derived from the incorporated silver nanoparticles.

The elemental composition and spatial distribution of the electrospun PCL/PEO dc-AgNP nanofibers were analyzed by SEM–EDS, and the results are presented in Figure 5. The EDS spectrum (Figure 5C) confirmed the presence of silver within the membrane through the characteristic Ag-Lα signal around ~3 keV, commonly used as the diagnostic peak for silver detection in nanostructured materials [46,47,48]. A strong oxygen (O-K) signal was also detected, corresponding to the ester and ether groups present in the PCL and PEO polymer chains. SEM–EDS analysis is frequently employed in electrospun systems containing AgNPs to verify the presence of silver, estimate its relative content, and evaluate its distribution within the fibrous network [45,48,49].

Elemental mapping (Figure 5A) revealed that Ag signals are distributed as discrete points along the fibers, indicating that the nanoparticles are dispersed throughout the electrospun matrix rather than concentrated in localized aggregates. The oxygen signal was uniformly distributed across the membrane, consistent with the chemical composition of the PCL/PEO blend. Similar studies report silver contents typically ranging from 1 to 7 wt% in electrospun fibers with moderate nanoparticle loading [50,51].

Overall, the homogeneous distribution of Ag observed in the mapping analysis suggests that the electrospinning process enabled effective dispersion of the nanoparticles within the fibrous scaffold, which is advantageous for antimicrobial applications because it promotes greater exposure of silver nanoparticles on the fiber surface.

#### 3.5.3. Thermal Properties

**Figure 6:**
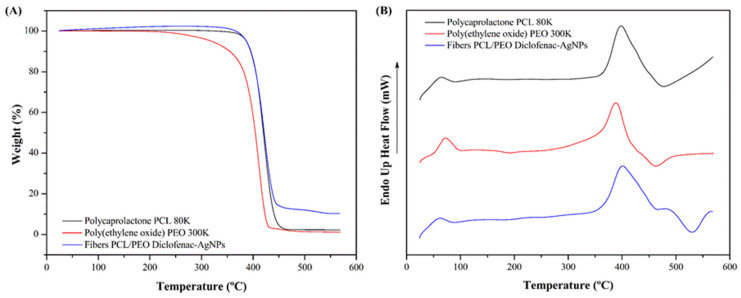
(**A**) TGA and (**B**) DSC profiles of PCL (black line), PEO (red line) and PCL/PEO dc-AgNP electrospun fibers (blue line).

The thermal behavior of the electrospun systems is summarized in Table 5, while the representative TGA and DSC thermograms are presented in Figure 6. These analyses provide insight into the crystalline organization and thermal stability of the individual polymers and the nanocomposite membrane.

The thermal characterization revealed distinct crystallization behaviors associated with polymer composition and nanoparticle incorporation. Neat PCL (80 kDa) exhibited a melting enthalpy (ΔH_m_) of 12.75 J/g and a melting temperature (T_m_) of 64.76 °C, corresponding to a crystallinity degree (C_x_) of 9.16%, which is consistent with partially crystalline electrospun PCL structures. The rapid solvent evaporation and extensional forces inherent to electrospinning typically restrict lamellar growth, resulting in reduced crystallinity compared to bulk materials. In agreement with previous reports, electrospun PCL generally preserves T_m_ values close to bulk (~56–61 °C), while variations in ΔH_m_ and crystallinity are mainly attributed to processing conditions and structural confinement effects [52,53,54]. In contrast, PEO (300 kDa) fibers showed a substantially higher ΔH_m_ (43.87 J/g) and crystallinity (21.61%), together with a higher T_m_ (71.91 °C), reflecting the stronger intrinsic crystallization tendency of PEO and its ability to reorganize into more ordered crystalline domains during fiber solidification. Consistent with the literature, electrospun PEO typically retains melting behavior like its bulk counterpart, indicating that the electrospinning process does not significantly alter its phase transition properties [55]. Furthermore, PEO-based electrospun systems commonly exhibit a single degradation stage and maintain thermal stability well above application temperatures, even after incorporation of functional additives [56,57,58]. The PCL/PEO dc-AgNP composite fibers displayed intermediate thermal behavior, with ΔH_m_ of 16.30 J/g, T_m_ of 62.09 °C, and C_x_ of 11.70%. The slight decrease in T_m_ relative to neat PCL suggests partial disruption of crystalline packing due to polymer blending and nanoparticle incorporation. The moderate increase in crystallinity compared to pure PCL may indicate heterogeneous nucleation induced by AgNPs, which can promote crystal initiation while limiting lamellar perfection. Similar behavior has been reported in electrospun polymer systems containing additives, where crystalline organization is modulated without significant changes in melting transitions [59,60].

Thermogravimetric analysis showed high thermal stability for all systems (Td > 410 °C), with the composite (421.83 °C) maintaining thermal resistance comparable to neat PCL (422.70 °C), confirming that nanocomposite incorporation preserves structural integrity. In line with previous studies, electrospun PCL systems typically exhibit a single main degradation stage with Tmax values between ~380 and 435 °C, and the presence of additives may slightly shift degradation temperatures without compromising overall stability [52,61].

#### 3.5.4. Attenuated Total Reflectance FTIR (ATR–FTIR)

**Figure 7:**
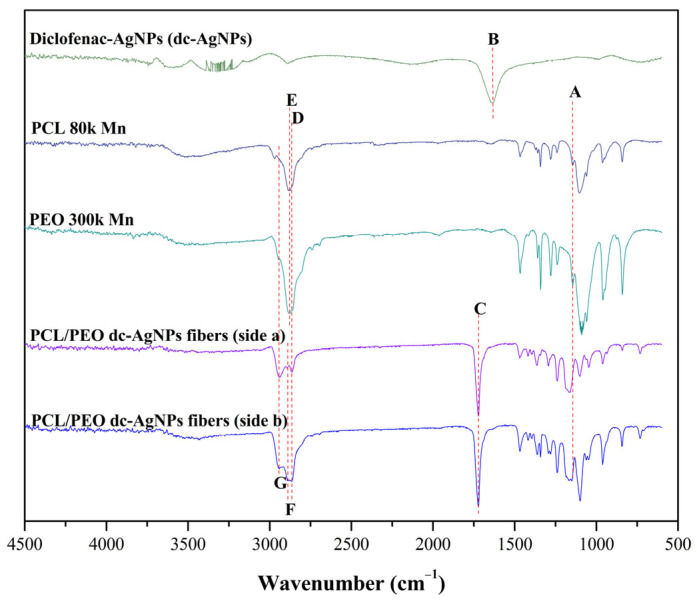
ATR–FTIR spectra of dc–AgNPs, PCL (80 k Mn), PEO (300 k Mn), and electrospun PCL/PEO dc-AgNP nanofibers (side a and side b), highlighting the characteristic absorption bands associated with the polymeric matrix and nanocomposite components. Band assignments: **A**, asymmetric stretching of ether C–O–C bonds; **B**, C–O stretching; **C**, carbonyl C=O stretching; **D**–**G**, C–H stretching vibrations.

The ATR–FTIR spectra of dc-AgNPs, raw dry PCL (80 kDa), raw dry PEO (300 kDa), and multilayer PCL/PEO dc-AgNP electrospun fibers obtained from both sides of the membrane are shown in Figure 7. The spectra of raw PCL and PEO were included as polymer references and do not correspond to electrospun fibers. The ester carbonyl stretching vibration of PCL is commonly reported around 1722 cm^−1^ and represents a diagnostic absorption of the polyester backbone, particularly in PCL-based electrospun fibers [25,62]. However, in the raw dry PCL reference spectrum shown in Figure 7, band C is not clearly resolved under the spectral conditions used. In contrast, the absorption in this region is more evident in the PCL/PEO dc-AgNP electrospun fibers, supporting the presence of PCL within the multilayer membrane. Additionally, the asymmetric C–O–C stretching vibration of the ester group appears at 1150.9 cm^−1^ (band A), consistent with semicrystalline aliphatic polyesters [63]. The bands located at 2865.7–2940.9 cm^−1^ (bands D–G) correspond to symmetric and asymmetric C–H stretching vibrations from methylene groups, confirming the aliphatic nature of PCL [25].

For PEO, the spectrum is dominated by strong ether (C–O–C) stretching vibrations in the 1100–1150 cm^−1^ region, overlapping partially with PCL signals in the composite fibers [64]. The higher crystallinity of PEO is reflected in the intensity and sharpness of these ether-related bands. In the dc-AgNP spectrum, the band at 1636.3 cm^−1^ (band B) is attributed to C–O stretching and aromatic contributions from diclofenac, indicating successful surface functionalization of the nanoparticles [65]. In the composite fibers, the coexistence of the ester carbonyl peak (1722 cm^−1^) and the ether-related absorptions confirm the physical blending of PCL and PEO within the multilayer structure. No significant peak shifts or new absorption bands were detected, suggesting the absence of covalent bond formation between polymers and nanoparticles. However, slight variations in band intensity between side a and side b indicate surface compositional differences, likely due to the multilayer deposition sequence. Overall, the ATR–FTIR results corroborate the structural integrity of both polymers and confirm the successful incorporation of dc-coated AgNPs without chemical degradation.

#### 3.5.5. Mechanical Properties

**Figure 8:**
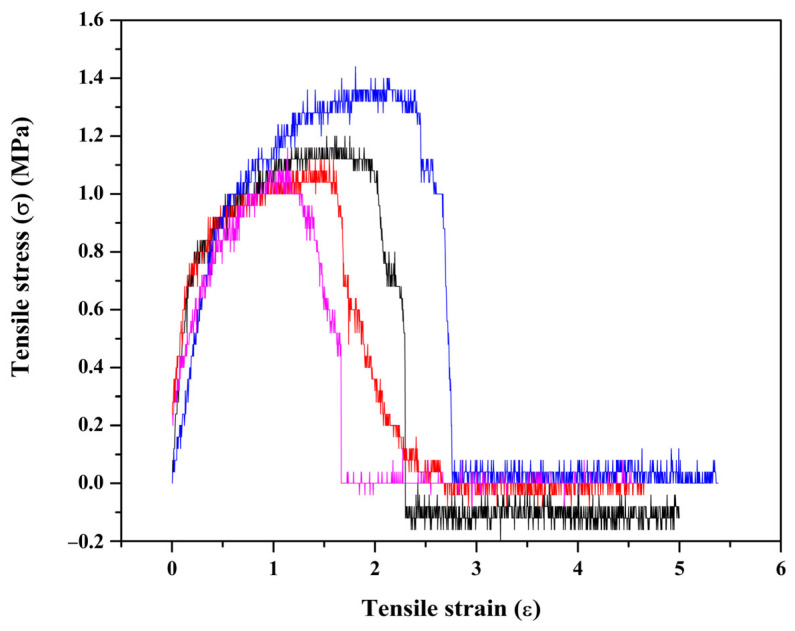
Stress–strain curves of PCL/PEO dc-AgNP electrospun membranes obtained from tensile testing. Curves correspond to four independent membrane specimens.

The mechanical properties of the electrospun PCL/PEO dc-AgNP membrane are summarized in Table 6, and the corresponding stress–strain curves are shown in Figure 8. Overall, the membrane exhibited a flexible and mechanically stable behavior, with high deformation capacity before failure.

The elongation at break of the membrane reached 145.6 ± 28.3%, indicating a highly ductile material capable of sustaining substantial deformation before failure. Such elongation values are characteristic of electrospun matrices composed of semicrystalline polymers like PCL, particularly when blended with more flexible polymers such as PEO or gelatin [66,67,68].

The tensile strength of the electrospun membrane was 1.2 ± 0.1 MPa, reflecting moderate mechanical strength combined with high flexibility. This strength is higher than that of highly porous random PEO/PCL membranes and within the lower range of some pure PCL membranes (0.8–2.5 MPa) [59]. The presence of diclofenac-coated silver nanoparticles may slightly alter the polymer chain organization within the fibers, potentially contributing to a slight reduction in tensile strength compared to pure polymer systems, while maintaining structural integrity.

The Young modulus of the membrane was 2.6 ± 1.3 MPa, indicating a relatively compliant and elastic material. This modulus falls within the lower range typically reported for electrospun PCL scaffolds, which generally exhibit Young’s modulus values between approximately 2 and 10 MPa depending on fiber morphology, porosity, and electrospinning parameters. The reduced stiffness observed in the PCL/PEO dc-AgNP fibers likely reflects the plasticizing effect of PEO and the structural heterogeneity introduced by nanoparticle incorporation [69,70,71]. Overall, the obtained mechanical properties suggest that the nanocomposite membrane presents adequate flexibility and mechanical stability for potential biomedical applications requiring conformable and mechanically resilient fibrous materials.

### 3.6. Antibacterial Activity of Diclofenac-AgNP Fiber

The antimicrobial activity of the electrospun PCL/PEO dc-AgNP nanofibers was evaluated using two complementary assays, the AATCC 100 contact killing test and the ASTM E2149 dynamic shake flask test, and the quantitative results are summarized in Table 7. Overall, the nanocomposite fibers exhibited microorganism-dependent antimicrobial effects, with generally higher activity observed under direct contact conditions.

In the contact killing assay, moderate antimicrobial reductions were observed after 1 h of contact between the microorganisms and the electrospun fibers. Both *P. aeruginosa* and *S. aureus* showed 0.4 log reductions, indicating a modest decrease in viable bacterial cells upon direct interaction with the nanofibrous surface. A slightly higher antimicrobial response was observed for *E. coli*, which exhibited a 0.7 log reduction, suggesting greater susceptibility of this Gram-negative bacterium to the nanocomposite surface. The most pronounced antibacterial effect was detected against *S. mutans*, with a 2.6 log reduction, indicating a substantial reduction in bacterial viability after exposure to the fibers. This higher sensitivity may be associated with differences in cell wall structure and adhesion mechanisms that facilitate stronger interactions with the nanofibrous matrix. In contrast, the antifungal activity against *C. albicans* remained limited, with a 0.4 log reduction, suggesting that the fungal cell wall may provide greater resistance to the antimicrobial components present in the fibers.

In the dynamic shake flask assay, the antimicrobial activity was generally lower, with log reductions of 0.1 for *S. aureus*, *E. coli*, and *S. mutans*, and 0.3 for *C. albicans*. A higher reduction of 0.9 log was observed for *P. aeruginosa*. The reduced antimicrobial effect under dynamic conditions suggests that the antimicrobial performance of the PCL/PEO dc-AgNP fibers is largely contact-dependent, likely mediated by direct interactions between microbial cells and the nanofiber surface where silver nanoparticles and diclofenac are present. Silver acts through direct interactions with the cell envelope (membrane damage, binding to thiol groups, respiratory disruption) and local generation of reactive oxygen species, typically following the release of Ag^+^ from nanoparticles near the electrospun fibers’ surface [72,73,74]. These findings are consistent with previous studies reporting that electrospun nanocomposite scaffolds containing silver nanoparticles often display enhanced antimicrobial activity through surface contact rather than through diffusion-based mechanisms. In these cases, the kinetics and amount of released Ag^+^ are correlated with bacterial inhibition, indicating that ion diffusion into the surrounding medium is crucial, especially for killing bacteria not directly attached to the fiber [75,76,77].

## 4. Conclusions

The functionalization of silver nanoparticles (AgNPs) with stabilizing ligands is a determinant of their biological activity, modulating their physicochemical properties, colloidal stability, and subsequent interfacial interactions within biological systems in a coordinated manner. Among the nanoformulations evaluated, diclofenac-stabilized AgNPs (dc-AgNPs) showed superior broad-spectrum antimicrobial efficacy, characterized by significantly lower minimum inhibitory and bactericidal concentrations (MICs/MBCs) against a diverse panel of Gram-positive, Gram-negative, and anaerobic pathogens, as well as *Candida albicans*. This potent bioactivity suggests a synergistic interaction between the silver metal core and the pharmacological ligands, particularly diclofenac and ketorolac, providing a strategic framework for the rational design of multifunctional bioactive nanocomposites. Furthermore, in vitro biocompatibility assessments revealed a nuanced, dose-dependent cytotherapeutic window, with fibroblast lineages exhibiting greater sensitivity compared to MG-63 cells and mesenchymal stem cells (MSCs). This underscores the critical need for cell-type-specific dose optimization in clinical applications. Successful encapsulation of dc-AgNPs in electrospun PCL/PEO arrays yielded a hierarchical nanofibrous architecture that effectively links mechanical structural integrity with localized antimicrobial functionality. Taken together, these findings highlight the fundamental role of ligand engineering in modulating nanoparticle performance and validate the integration of functionalized AgNPs into electrospun scaffolds as a robust platform for treating recalcitrant biofilm-associated wound infections.

## Figures and Tables

**Table 1:** Summary of HET-CAM score ranges used in irritation classification.

HET-CAM Score Range	Irritation Category
0–0.9	Nonirritant or Practically None
1–4.9	Weak or Slight Irritation
5–8.9 or 5–9.9	Moderate Irritation
9–21 or 10–21	Strong or Severe Irritation

From Luepke [23] and Kalweit et al. [24].

**Table 2:** MIC values (mg/L) for NaCitr-AgNPs, cyst-AgNPs, kt-AgNPs, and dc-AgNPs, evaluated against *S. aureus*, *P. aeruginosa*, *E. coli*, *S. mutans*, *C. albicans*, *Porphyromonas gingivalis*, and *Fusobacterium nucleatum*.

MIC (mg/L)						
Ligand-Stabilized Silver Nanoparticles	*P. aeruginosa*	*E. Coli*	*S. mutans*	*S. aureus*	*C. albicans*	*Porphyromonas gingivalis*, *Fusobacterium nucleatum*
NaCitr-AgNPs	300	300	150	75	75	75 (*F. nucleatum*) 300 (*P. gingivalis*)
Cyst-AgNPs	150	75	150	150	37.5	300 (*F. nucleatum*) 150 (*P. gingivalis*)
kt-AgNPs	37.5	75	150	150	9.4	150 (*F. nucleatum*) 75 (*P. gingivalis*)
dc-AgNPs	18.8	75	37.5	18.8	4.7	75 (*F. nucleatum*) 37.5 (*P. gingivalis*)

**Table 3:** MBC values (mg/L) for NaCitr-AgNPs, cyst-AgNPs, kt-AgNPs, and dc-AgNPs evaluated against *S. aureus, P. aeruginosa, E. coli, S. mutans, C. albicans, Porphyromonas gingivalis,* and *Fusobacterium nucleatum*.

MBC (mg/L)						
Ligand-Stabilized Silver Nanoparticles	*P. aeruginosa*	*E. Coli*	*S. mutans*	*S. aureus*	*C. albicans*	*Porphyromonas gingivalis*, *Fusobacterium nucleatum*
NaCitr-AgNPs	>300	>300	>300	>300	>300	150 (*F. nucleatum*) >300 (*P. gingivalis*)
Cyst-AgNPs	>300	>300	>300	>300	>300	>300 (*F. nucleatum*) >300 (*P. gingivalis*)
kt-AgNPs	150	150	>300	150	75	>300 (*F. nucleatum*) *150* (*P. gingivalis*)
dc-AgNPs	75	75	150	75	18.8	150 (*F. nucleatum*) *75* (*P. gingivalis*)

**Table 4:** Mean diameter and morphology index of the obtained electrospun fibers.

Electrospun Fibers	Fiber Thickness (µm)			Morphology Index
PCL 14%	3.212 ^a^	±	1.577	0.65
PEO 10–dc-AgNPs	0.343 ^b^	±	0.073	0.85

**a** and **b** express statistically significant differences, with the formation of subsets.

**Table 5:** Thermal properties of PCL, PEO and PCL/PEO Diclofenac-AgNP electrospun fibers.

Samples	∆H_m_	T_m_	C_x_	T_d_
	(J/g)	(°C)	(%)	(°C)
Polycaprolactone PCL 80 k Mn	12.75	64.76	9.16	422.70
Poly(ethylene oxide) PEO 300 k Mn	43.87	71.91	21.61	410.75
PCL/PEO dc-AgNPs fibers	16.30	62.09	11.70	421.83

**Table 6:** Mechanical properties of PCL/PEO dc-AgNPs in quadruplicates of electrospun membranes.

Samples	Elongation at Break (%)	Tensile Strength (MPa)	Young’s Modulus
PCL/PEO dc-AgNPs 1	152.4	1.2	4.3
PCL/PEO dc-AgNPs 2	135.2	1.1	2.7
PCL/PEO dc-AgNPs 3	180.8	1.4	2.1
PCL/PEO dc-AgNPs 4	113.9	1.2	1.3
Mean ± SD	145.6 ± 28.3	1.2 ± 0.1	2.6 ± 1.3

**Table 7:** Antimicrobial activity in Log_reduction_ of PCL/PEO dc-AgNP electrospun fibers against *P. aeruginosa*, *S. aureus*, *E. coli*, *S. mutans* and *C. albicans* by AATCC 100 contact killing test and the ASTM E2149 dynamic shake flask test.

	Contact Killing			Shake Flask		
	Fiber T_0_	Fiber T_1_	Log_reduction_	Microorganism T_5_	Fiber T_5_	Log_reduction_
	CFU/mL	CFU/mL		CFU/mL	CFU/mL	
*P. aeruginosa*	2.57 × 10^−1^	9.5 × 10^−2^	0.4	1.65 × 10^−3^	2.17 × 10^−4^	0.9
*S. aureus*	5.62 × 10^0^	2.17 × 10^0^	0.4	9.95 × 10^−6^	8.13 × 10^−6^	0.1
*E. coli*	2.85 × 10^−1^	5.83 × 10^−2^	0.7	5.47 × 10^−4^	4.45 × 10^−4^	0.1
*S. mutans*	2.51 × 10^3^	1.15 × 10^1^	2.6	5.17 × 10^−4^	3.83 × 10^−4^	0.1
*C. albicans*	2.43 × 10^1^	1.05 × 10^1^	0.4	2.88 × 10^−4^	1.47 × 10^−4^	0.3

## Data Availability

The original contributions presented in this study are included in the article. Further inquiries can be directed to the corresponding author.

## Abbreviations

The following abbreviations are used in this manuscript:

AATCC	American Association of Textile Chemists and Colorists
AgNO_3_	Silver nitrate
AgNPs	Silver nanoparticles
ASTM	American Society for Testing and Materials
ATR–FTIR	Attenuated total reflectance FTIR
BHI	Brain heart infusion
CFU	Colony-forming units
CLSI	Clinical Laboratory Standards Institute
Cyst	Cysteine
Cyst-AgNPs	Cysteine-stabilized silver nanoparticles
Cx	Degree of crystallinity
dc	Diclofenac
dc-AgNPs	Diclofenac-stabilized silver nanoparticles
DMEM	Dulbecco’s Modified Eagle Medium
DSC	Differential scanning calorimetry
ECM	Extracellular matrix
EDS	Energy-dispersive X-ray spectroscopy
FTIR	Fourier-transform infrared spectroscopy
HET-CAM	Hen’s Egg Test—Chorioallantoic Membrane
ICT	Interfacial charge transfer
IS	Irritation score
kt	Ketorolac
kt-AgNPs	Ketorolac-stabilized silver nanoparticles
MBC	Minimum bactericidal concentration
MHB	Mueller–Hinton broth
MIC	Minimum inhibitory concentration
MG-63	Human osteosarcoma cell line
MI	Morphology index
MSCs	Mesenchymal stem cells
MTT	3-(4,5-dimethylthiazol-2-yl)-2,5-diphenyltetrazolium bromide assay
NaBH_4_	Sodium borohydride
NaCitr	Sodium citrate
NaCitr-AgNPs	Sodium citrate-stabilized silver nanoparticles
NSAID	Nonsteroidal anti-inflammatory drug
PBS	Phosphate-buffered saline
PCL	Polycaprolactone
PEO	Poly(ethylene oxide)
ROS	Reactive oxygen species
SEM	Scanning electron microscopy
SEM–EDS	Scanning electron microscopy with energy-dispersive X-ray spectroscopy
SPR	Surface plasmon resonance
Td	Degradation temperature
TEM	Transmission electron microscopy
Tg	Glass transition temperature
TGA	Thermogravimetric analysis
Tm	Melting temperature
TSA	Tryptic soy agar
TSB	Tryptic soy broth
UV–Vis	Ultraviolet–visible spectroscopy
ζ (zeta potential)	Zeta potential
ΔH/ΔHm	Enthalpy of fusion
α-MEM	Alpha Minimum Essential Medium
